# The Beat

**Published:** 2010-04

**Authors:** Erin E. Dooley

## Foodborne Illness Costs: No Small Potatoes for the United States

The Produce Safety Project of The Pew Charitable Trusts estimated in March 2010 that foodborne illnesses cost the United States $152 billion each year and each citizen an average of $1,850 per case. The report, available at www.makeourfoodsafe.org, based its estimate on medical costs as well as costs due to lost life expectancy, pain and suffering, and functional disability. The CDC estimates more than 76 million new cases of foodborne illness resulting in 5,000 deaths and 325,000 hospitalizations occur each year in the United States.

**Figure:**
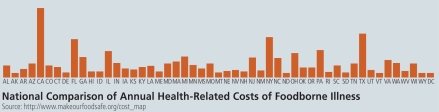
**National Comparison of Annual Health-Related Costs of Foodborne Illness** Source: http://www.makeourfoodsafe.org/cost_map

## TSCA Information Now Free Online

The EPA announced 15 March 2010 it will now provide free online access to the Toxic Substances Control Act Chemical Substance Inventory, which provides data on thousands of industrial chemicals. Until now, this consolidated set of information was available only by purchase. The move is part of the agency’s stated priority of making chemical information more accessible to the public and follows a January announcement that the EPA is seeking to reduce some confidentiality claims on the identity of chemicals (read more about chemical confidentiality on p. A168 of this issue). The inventory is available at http://www.epa.gov/oppt/newchems/pubs/invntory.htm.

**Figure:**
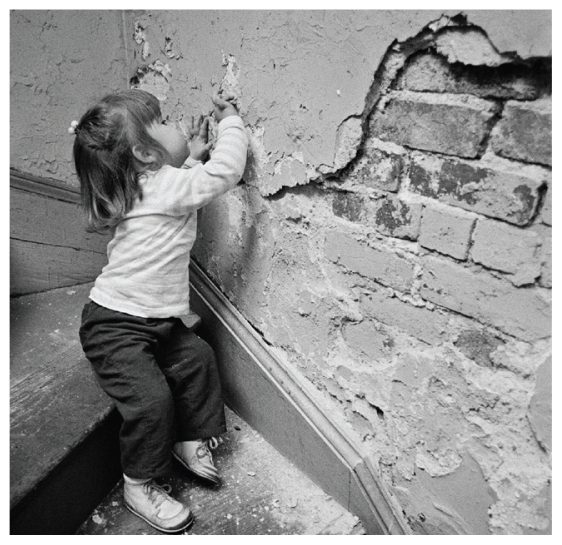
Paint and contaminated dust are major sources of lead exposure in U.S. children.

## New Lead Paint Rule Takes Effect

Effective 10 April 2010 all renovations of housing constructed before 1978 and of child-occupied facilities (such as schools) must be performed by certified renovators using specific lead-safe work practices. “Renovation” is defined broadly under the EPA rule to include window repair, weatherization, and modification of painted doors. The new regulation goes beyond earlier tenant notification stipulations by requiring that renovators post warning signs at the remodeling site to inform workers and occupants of lead hazards. Contractors also must now follow lead dust containment and waste management procedures. The EPA provides more information for contractors, certification trainers, homeowners, and landlords at www.epa.gov/getleadsafe.

## UNEP Offers E-Waste Predictions, Guidance

The world’s stockpile of e-waste—discarded computers, mobile phones, and other electronic devices—is growing by an estimated 40 million tons per year with little sign of stopping. In *Recycling—From E-Waste to Resources*, released 22 February 2010, UNEP estimates the number of discarded computers shipped to some developing countries could increase by as much as 500% by 2020. The informal recycling of electronics is a lucrative but highly hazardous cottage industry in many developing countries. The UNEP report therefore offers guidance for countries to build successful and safer e-waste management systems.

## Review of Environmental Factors in Malaria’s Spread

A review by Luis Fernando Chaves and Constantianus Koenraadt in the March 2010 *Quarterly Review of Biology* assesses the factors contributing to increases in malaria cases worldwide. The researchers report that climate change, human migration, and land-use changes all are causing malaria to spread into highland areas of East Africa, Indonesia, Afghanistan, and elsewhere. They systematically show how climate affects multiple biological components of malaria transmission and highlight the need for research to better understand the transmission dynamics of this disease and how to sustainably control or eliminate it.

## PAHs: Pathways to Waterways

Polycyclic aromatic hydrocarbons (PAHs), chemicals released during combustion of biomass and fossil fuels, are ubiquitous in the environment. Lisa Rodenburg and colleagues undertook a 4-year study to identify the primary routes by which PAHs end up in New York/New Jersey Harbor. Their findings, reported in the March–April 2010 *Journal of Environmental Quality*, show stormwater runoff was the main pathway, contributing about half the harbor’s PAH load, and atmospheric deposition was an important contributor of smaller PAH compounds. The results suggest that minimizing the flow of PAHs into waterways may require tweaking stormwater management plans to control runoff.

**Figure:**
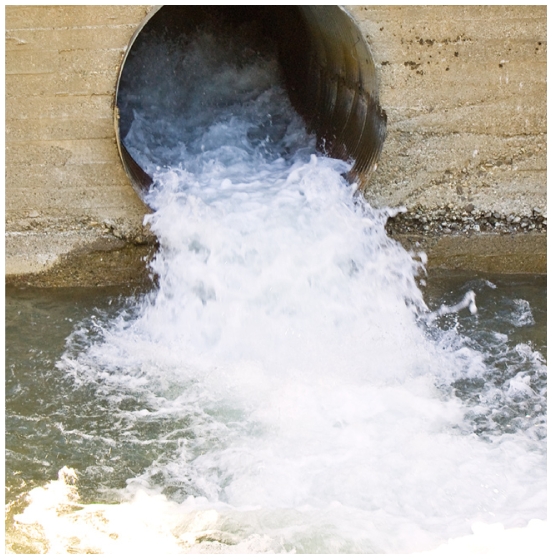
Stormwater runoff is a major route by which PAHs enter waterways.

